# Predicting patient‐reported quality of vision through multifocal optics

**DOI:** 10.1111/opo.13556

**Published:** 2025-07-18

**Authors:** Pete Kollbaum, Dawn Meyer, Nitya Murthy, Javier Gantes‐Nunez, Matt Jaskulski, Martin Rickert

**Affiliations:** ^1^ School of Optometry Indiana University Bloomington Indiana USA

**Keywords:** contact lenses, image quality, myopia, point‐spread function, presbyopia

## Abstract

**Purpose:**

To explore the agreement between computationally modelled point‐spread functions (PSFs) and multifocal soft contact lens wearers' descriptions of image quality.

**Methods:**

In Phase 1, participants were fitted with a single vision soft contact lens (SCL) in the left eye, and four different multifocal (MF) SCLs in the right eye in randomised order. Aberrometry measures of the MFSCL‐wearing right eye were collected while participants viewed a point source monocularly using their left eye and drew their perception of the source. The predicted right eyes' PSFs were computed from the aberrometry data using a geometrical optics PSF calculation. In Phase 2, participants completed a Likert item questionnaire designed to identify salient features in the PSFs. Consensus among the participants about these features was assessed quantitatively using Yule's *Q*. Participants also rated the similarity between pairs of PSFs. Non‐metric multidimensional scaling (MDS) and Procrustes transformation analyses were used to derive and compare image similarity spaces for the drawn and computed PSFs, respectively. Lastly, two observers completed an image matching task, in which they selected the best‐matching drawn PSF for each computed PSF.

**Results:**

Significant similarities between computed and hand‐drawn PSFs were demonstrated both qualitatively and quantitatively. Inter‐rater agreement for image features was statistically significant (Yule's *Q* = 0.63; *p* < 0.05). Stress values for three‐dimensional MDS configurations were <0.1, indicating excellent correspondence between MDS configurations and observed similarity ratings. Procrustes analysis confirmed strong concordance between hand‐drawn and computed 3D configurations (congruence coefficient = 0.90). Image matches were inconsistent with random guessing (*p* < 0.001).

**Conclusions:**

Results indicate good agreement between the PSF image quality drawn by participants and that predicted from aberrometry measures. This study has demonstrated potential for leveraging the correspondence between subjectively perceived and computationally derived representations of image quality in both clinical and contact lens research and development processes.

**Key points**

- There was excellent agreement (e.g., shape) between computed geometric and hand‐drawn point‐spread functions.
- Participant‐drawn images provide a reliable method of capturing image quality.
- Monocular computational optical modelling performs well in predicting observer image quality.

## INTRODUCTION

In order to correct a patient's vision optimally, there is a strong rationale for both clinicians and lens manufacturers to be able to predict the optical impact of placing a soft contact lens (SCL) on an eye.^1^ This is especially the case for lenses designed for myopia control and presbyopic correction, which constitute an increasing portion of the worldwide contact lens market.^2^, ^3^ These multifocal and/or dual focus lenses are based on various representative optical design principles including multiple alternating zones of lens power (e.g., MiSight 1‐day; CooperVision, Inc., coopervision.com), centre‐near surround (e.g., Biofinity Multifocal; CooperVision, Inc., coopervision.com) or aspheric (e.g., AirOptix Multifocal; Alcon, Inc., alcon.com) designs. These design features intentionally introduce peripheral defocus and/or create multiple focal points, both of which may impact image quality (IQ) and visual performance.^4^ While clinical testing methods, such as visual acuity^5^ and contrast sensitivity^6^ are often used to provide estimates of the image quality attained when a lens is placed on an eye,^7^, ^8^ computational models can now also provide such estimates.^9^, ^10^, ^11^, ^12^ Image quality (IQ) metrics have been shown to correlate highly with perceived visual acuity^13^, ^14^ and have been used to make predictions of the best contact lens design.^15^ This work, however, was related to eyes which were either uncorrected or corrected using optics which were not multifocal. Further, objective measures of image quality, such as visual acuity, do not adequately represent the image quality attainable with multifocal lenses.^16^, ^17^ Specifically, multifocal optics can introduce morphological elements into point‐spread functions (PSFs) such as rings, patches or patterns secondary to the PSF core that can be left unaccounted for by traditional measures of image quality. The primary objective of this study was to address this gap by assessing the agreement in shape between computationally modelled PSFs and the shape of the subject‐reported descriptions of image quality in eyes wearing multifocal SCLs, representing a sample of the range of commercially available optical designs.

## METHODS

The experimental protocols followed the ethical principles as outlined in the tenets of the Declaration of Helsinki and were approved by the Indiana University Institutional Review Board (IRB) research ethics committee. Prior to participation, all participants provided written informed consent after being fully briefed on the study's purpose, procedures, potential risks and their right to withdraw at any time. The study was performed in two phases.

### Phase 1

The primary aim was to obtain both subjective and objective IQ data from a sample of habitual SCL wearers while wearing four commercially available multifocal soft contact lenses (MFSCL). A single vision (control) best‐correction SCL fitted on the left eye served as the contralateral comparator for each MFSCL fitted in the right eye.

#### Participants

A total of 10 participants (six females and four males) with a mean (SD) age of 29.0 (6.5) years were enrolled in Phase 1. All participants were experienced, habitual SCL wearers with good ocular health, best‐corrected Snellen visual acuity of at least 6/6 and a cylinder <0.75 D in each eye.

#### Lenses

Information regarding the technical specifications of the lenses used in this study is provided in Table 1.

**TABLE 1:** Technical specifications for the five contact lens types used in Phase 1.

	Control lens	Test lens 1	Test lens 2	Test lens 3	Test lens 4
Lens	Proclear 1‐day	Biofinity Multifocal (+2.00 D add)	AirOptix Multifocal (medium add)	MiSight 1‐day	NaturalVue 1‐day
Manufacturer	CooperVision, Inc., coopervision.com	CooperVision, Inc., coopervision.com	Alcon, Inc., alcon.com	CooperVision, Inc., coopervision.com	Visioneering Technologies, Inc., vti.com
Lens Design	Single Vision	Centre Distance	Centre near (Bi‐aspheric, adaptive minus power profile)	Dual focus Zonal aspheric	Extended depth of focus
Material	Omafilcon B	Comfilcon A	Lotrafilcon B	Omafilcon A	Etafilcon A
Base Curve/Diameter (mm)	8.6/14.2	8.6/14.0	8.6/14.2	8.7/14.2	8.3/14.5

*Note*: The manufacturer, lens design, material and base curve diameter are listed separately for each lens type.

#### Procedure

Prior to any testing and with participants best‐corrected bilaterally, instructions for the task were provided. To prime their attention to the task, participants were shown printed sample point source images including a range of possible attributes which may be visible during the experiment when they viewed the point source. These samples included an aberration‐free point, a point with arcs/curves/rings around its centre, a point with lines or spokes emanating from its centre, a point with a tail, etc. As participants viewed these sample images, the investigator provided special instruction to the participant to pay particular attention to the relative brightness of features, symmetry, orientation, number of features, etc., of the source they were going to observe. Sample point source images were created using custom software (Indiana Wavefront Analyzer, IWA) written in MATLAB (MathWorks, mathworks.com). Following instruction, participants were first fitted bilaterally with a single vision control SCL and over‐refracted. The left eye remained in the single vision control SCL while the right eye was fitted with each of the four different MFSCLs in a randomised order. Appropriate lens fit and centration was ensured via slit lamp biomicroscopy.

**FIGURE 1:**
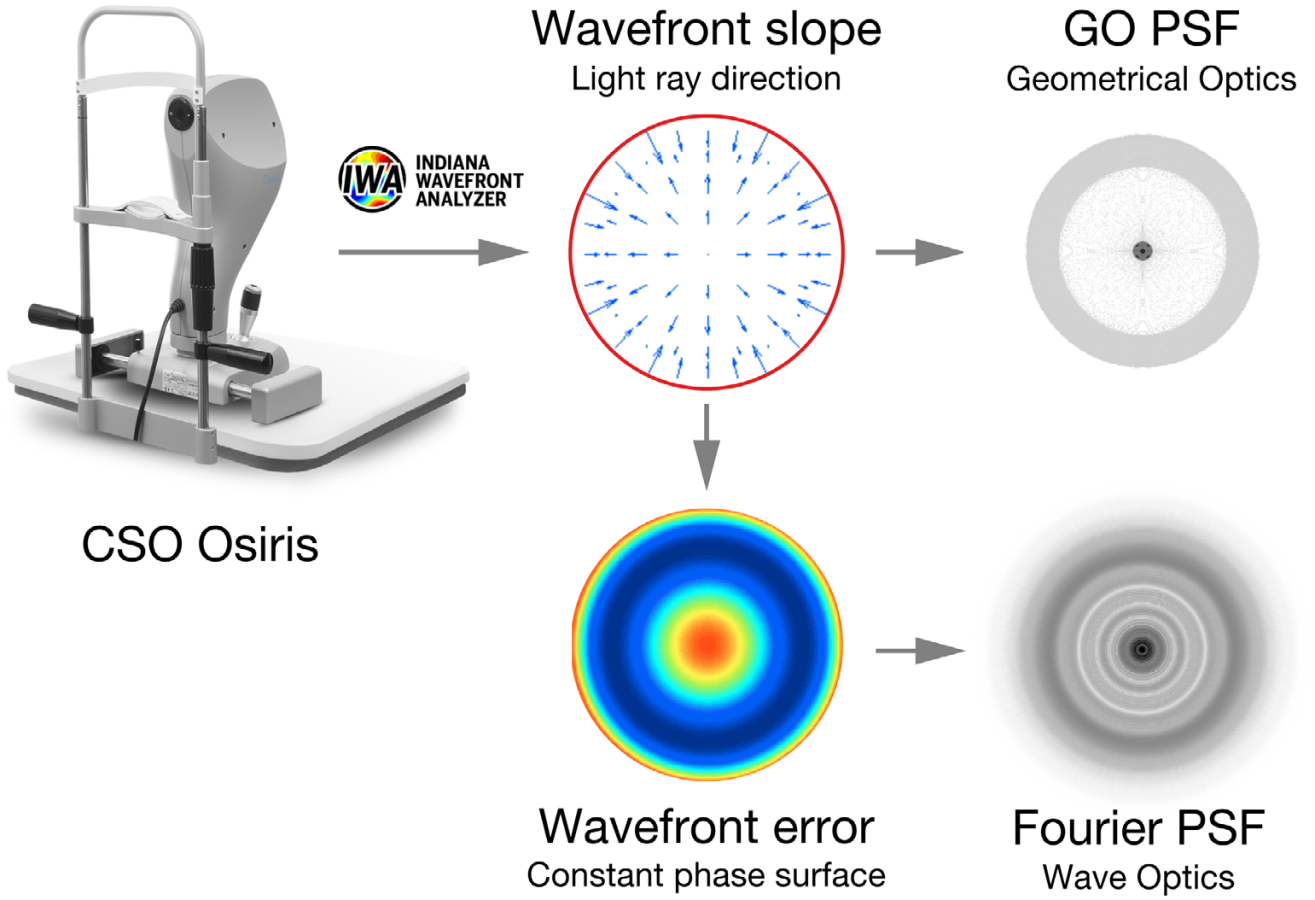
Schematic of data acquisition and wavefront analysis system used to derive geometric point‐spread functions (PSFs) from aberrometry data.

Participants viewed a green light‐emitting diode (LED) (570 nm, GL Spectris 1.0 Spectrometer, gloptic.com) point source of light at 4 m under low room illumination (1–5 lux) through the right eye corrected for distance viewing by the MFSCL, and relied on their left single vision control SCL wearing eye to provide a hand‐drawn representation and written description of the LED point source. Participants were instructed to fixate the LED point source and provide responses after allowing any post‐blink lens settling, in order to minimise off‐axis aberration or change in lens position resulting in differing appearance. Simultaneously, wavefront measurements were obtained using a validated pyramidal wavefront sensor Osiris (CSO, csoitalia.it)^18^ which was situated behind a beam splitter. The instrument nominally corrects any residual defocus to introduce a small point source of light into the eye for the measurement (and adds this back in for the analysis) and has a large dynamic range (~8.00 spherical and 10.00 D astigmatic defocus) to accommodate measurement of multifocal lenses.^18^ Wavefronts were measured across the full natural pupil and exported from the aberrometer for processing by custom software (IWA; Maple, LLC) written in MATLAB. Specifically, because wavefront measures were acquired during participant LED fixation, the optical measures quantify the same optics utilised during the time for which participants were making the image quality judgements. The aberrometer‐measured wavefront slopes were converted into geometrical optics (GO) PSFs by computing a histogram of light ray intersections with the image plane. The derivation and mathematical details have been described in detail elsewhere, resulting in a PSF representing a distant target.^19^, ^20^, ^21^ Figure 1 provides a schematic overview of the signal processing steps.

### Phase 2

The primary aim was to assess quantitatively how well participant‐drawn PSFs aligned with the computed GO PSFs. Multiple approaches were used. Specifically, from the 50 PSF image pairs collected in Phase 1, a subset of 15 pairs (i.e., 15 drawn PSFs each with the corresponding 15 GO PSFs) was randomly selected for testing in Phase 2, which comprised three psychometric tasks:

1. Feature assessment and agreement: Participants rated the 30 PSFs (15 hand‐drawn +15 computed) on their strength of agreement with 13 declarative statements (i.e., feature ‘descriptors’) using a 5‐point Likert scale (‘1 = Strongly disagree’ to ‘5 = Strongly agree’).
2. Similarity judgement: Participants rated all possible PSF image pairings on their perceived similarity using a 10‐point scale (‘1 = Extremely dissimilar’ to ‘10 = Identical’). Mean similarity ratings were analysed using non‐metric multidimensional scaling (MDS).
3. Direct image‐matching: Two participants selected the best match for each of the 15 GO PSFs from the corresponding set of 15 hand‐drawn PSFs.

Note that using a subset of the image pairs was necessary as evaluating all 50 pairs would have increased participant burden across tasks significantly. Specifically, the feature assessment and agreement task would have increased from 390 item responses (30 PSFs × 13 descriptors) to 1300 (100 PSFs × 13 descriptors), while the similarity judgement task would have increased from 225 pairwise comparisons (15^2^ pairs) to 2500 (50^2^ pairs), imposing excessive cognitive and time demands on participants. We verified by visual inspection that the sampled subset included a comparable range of features to the full image set to ensure representativeness.

Additional details for each task and analysis are described below.

#### Participants

A total of 14 volunteers (nine females and five males) with a mean (SD) age of 26.1 (1.3) years participated in the second phase of the study. None of these volunteers were participants in Phase 1, and all were naïve with respect to the image data and analysis collected during Phase 1 of the study. All tasks were administered, and responses were collected electronically using Qualtrics survey software (Qualtrics XM, qualtrics.com).

#### Procedures

##### Feature assessment and agreement

This task was designed to identify the most perceptually salient features and attributes in each of 15 hand‐drawn and corresponding GO PSFs using a predefined set of 13 feature descriptors developed through expert content validity analysis. The descriptors were presented as declarative statements:

1. There are arcs or curves.
2. There are features that resemble wheel spokes (e.g., lines radiating from the centre).
3. There are distinct bands or lines.
4. There are multiple spokes or tails emanating from the central section of this image.
5. There is a single quadrant/section with lines or spokes.
6. There are multiple quadrants/sections with lines or spokes.
7. There is a central core.
8. A central core makes up most of the image.
9. If you divide the image vertically, the left side looks like the right.
10. If you divide the image horizontally, the top looks like the bottom.
11. There is complexity. For this question, a simple image would have a few distinct features, and a complex image has many distinct features.
12. There is regularity. For this question, ‘regular’ means there is a consistent and predictable pattern of elements (e.g., a spiral), versus ‘irregular’ (e.g., a splat of paint).
13. The brightness or density is uniform throughout the image.

Note that the descriptors represent distinct aspects of shape that have been associated with perceived blur and image quality, including radial and linear distribution of light and the uniformity of blur across various image orientations.^22^ The final electronic questionnaire consisted of 390 5‐point Likert scale items, that is, 15 hand‐drawn PSFs + 15 GO PSFs × 13 feature descriptors. In addition to analysing the Likert categorical responses separately for each PSF, a group‐level analysis was performed to quantify the level of agreement between the features that participants identified in the geometrical version versus those that were identified in the hand‐drawn version. This analysis began by constructing two binary feature ‘agreement’ vectors, one for the GO PSF and the other for the drawn PSF. Each element in these vectors corresponded to one of the 13 feature descriptors listed above. Next, for each feature, the total percentage of participants who responded either ‘Agree’ or ‘Strongly Agree’ was calculated. If the combined exceeded 50%, a value of ‘1’ was assigned to that feature in the vector and ‘0’ otherwise. Then, Yule's *Q* was calculated for each pair, a non‐parametric measure of association between binary variables. Lastly, descriptive statistics (mean, standard deviation, minimum and maximum) were quantified across all 15 PSF pairs to summarise the estimated associations.

##### Similarity judgements

To complement the results obtained from the feature‐agreement task, three variants of a similarity ratings task were developed. In each variant, participants were presented with and rated the perceptual similarity of all possible unique and self‐pairings of the PSF images using a 1–10 similarity rating scale. To facilitate comparison of the two PSF image types, the geometric versions were inverted to black‐on‐white to match the hand‐drawn PSF appearance and scaled to approximately equate visual angles. Rating responses were collected in three separate blocks of trials:

1. In the Drawn‐to‐Drawn (D2D) block, participants compared and rated the similarity between all pairings of 15 hand‐drawn PSFs only.
2. In the Geometric‐to‐Geometric (G2G) block, participants compared and rated the similarity between all pairings of 15 GO PSFs only.
3. In the Drawn‐to‐Geometric (D2G) block, participants compared and rated the similarity between all pairings of 15 hand‐drawn PSFs and 15 GO PSFs.

A descriptive analysis was performed first to examine group‐averaged PSF image similarity ratings across the three different task conditions. The mean ratings from each condition were then arranged in matrix form, converted to dissimilarities and analysed using MDS, a multivariate statistical technique used to represent data in a low‐dimensional space, where the geometric configuration of points reflects the pairwise dissimilarities between individual stimuli.^23^, ^24^, ^25^, ^26^ One of the primary purposes of MDS is to visualise relationships or patterns among stimuli that might not be apparent in higher dimensional spaces. Consistent with a typical MDS approach, this analysis followed three key steps. First, comparative distances between stimuli were obtained from subjective ratings of similarity between drawn‐to‐drawn (D2D), geometric‐to‐geometric (G2G) and drawn‐to‐geometric (D2G) PSFs. Specifically, for each of the three comparison types, the group‐averaged similarity ratings were converted into dissimilarities in square symmetric matrix form by reverse scaling (i.e., *d*_*ij*_ = min(*s*_*ij*_) + max(*s*_*ij*_) − *s*_*ij*_) so that each cell value, *d*_*ij*_, quantified the perceived ‘distance’ between two images. The resulting matrix was input into a nonmetric MDS algorithm as implemented in the R package ‘smacof’ (version 2.1‐7) which uses iterative optimisation methods to minimise a global stress function (e.g., goodness‐of‐fit criterion) to generate a low‐dimensional spatial configuration where inter‐point distances directly corresponded with the estimated dissimilarities between PSF images.^27^, ^28^ Kruskal's stress‐1 function was used to quantify the total discrepancy between the observed dissimilarities and modelled distances. A stress value of zero indicates that the MDS configuration distances perfectly reproduced the input data while positive stress values reflect the degree to which the configuration distances deviate from those of the input data. While additional dimensions necessarily decrease or maintain the stress (never increase it), higher dimensionality can complicate meaningful interpretation. The determination of dimensionality used here was guided by a trade‐off between interpretability and model fit. Specifically, choice of dimensionality was based primarily on achieving sufficiently low stress, configuration interpretability and statistically significant permutation tests, which rejected the null hypothesis that the stress and configuration could have arisen from a random permutation of dissimilarities. To ensure robustness, this study also (1) explored the effect of randomising the initial configuration on resulting stress values, (2) assessed the stability of each MDS solution by bootstrapping a 95% confidence region on each image in the configuration space and (3) examined the configurations derived for additional dimensions. Finally, to examine whether the underlying spatial relationships between PSF images were preserved across the different MDS configurations, the configurations for D2D and G2G were compared to the D2G configuration using Procrustes transformation which removes irrelevant differences such as rotation, translation and dilation.^29^

##### Direct matching

Lastly, a direct, image‐matching task was performed to validate the correspondence between the 15 GO PSFs and 15 hand‐drawn PSFs. Two of the participants independently evaluated printed images from Phase 2's initial tasks, identifying which geometric PSF provided the closest perceptual match to each hand‐drawn PSF. This matching procedure provides an additional, objective measure of similarity that complements data from feature‐agreement and similarity rating tasks. Specifically, one can evaluate matching performance by comparing the observed proportion of correct matches to the expected proportion (i.e., probability) under the assumption of random guessing. The random guessing probability can be calculated by multiplying the binomial coefficient $\left(\frac{15}{k}\right)$ for the possible number of ways that *k* matches could be made from a group of *n* = 15 images times the derangement $!\left(n-k\right)$ of the remaining incorrect matches. Based on the resulting expression $\left(n-k\right)!\sum_{m=0}^{n-k}\frac{{\left(-1\right)}^{m}}{m!}$, it was noted that 0 and 1 matches were the most likely outcomes, each with a probability of 0.37. The probability decreases rapidly after two matches such that observing more than five matches by random guessing is extremely unlikely (*p* < 0.001), that is, one would expect to see five or more matches by chance alone in a thousand or more attempts.

## RESULTS

### Phase 1

#### Geometrical and hand‐drawn PSFs

**FIGURE 2:**
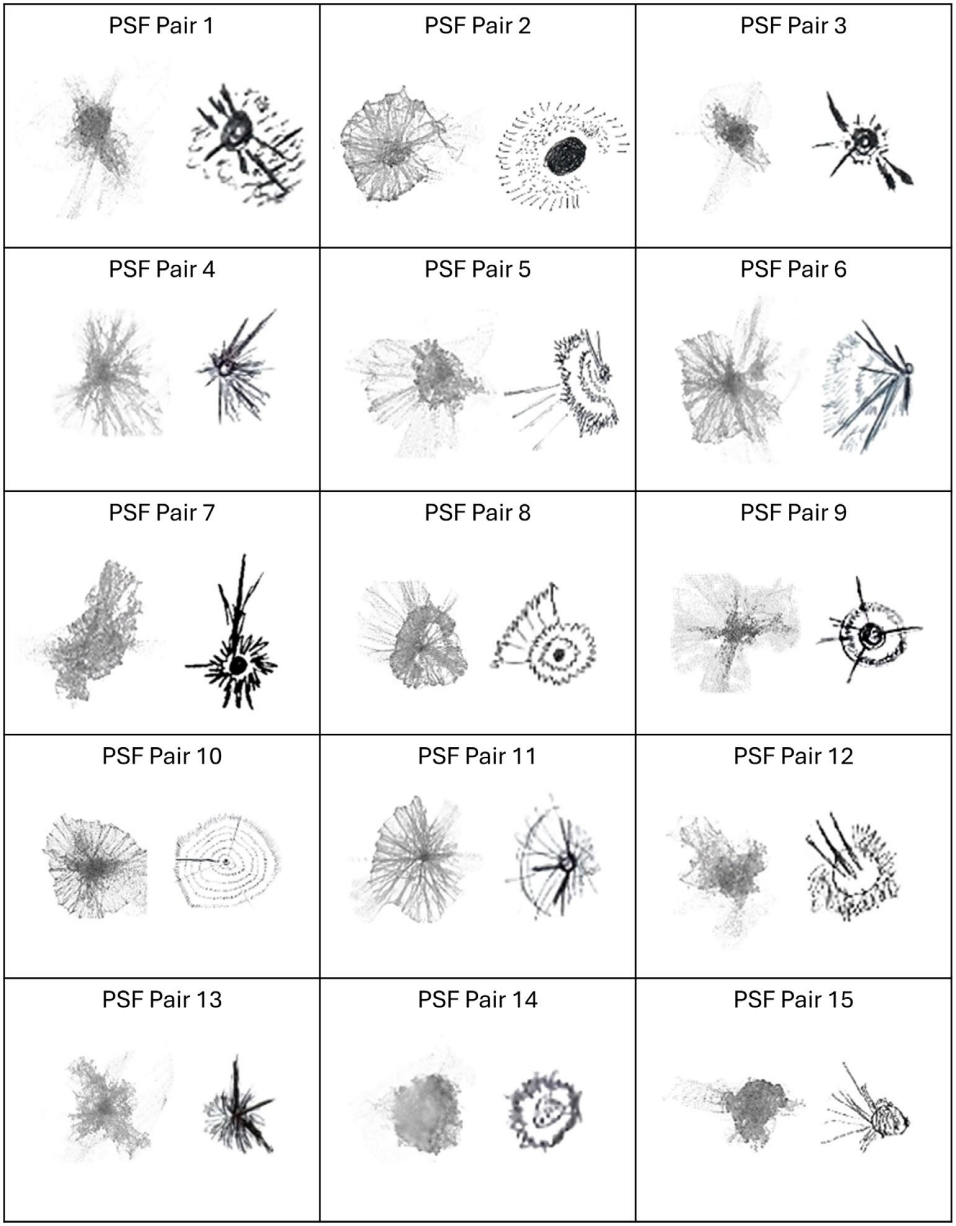
Side‐by‐side comparison of the 15 geometrical (left image in each cell) point spread functions (PSFs) and corresponding hand‐drawn (right image in each cell) PSFs used as stimuli in Phase 2. The geometrical version of the PSF was inverted to match the black ink (on a white background) presentation of the hand‐drawn version.

Recall that the primary aim of Phase 1 was to obtain both objective and subjective IQ data. Average pupil diameters of the participants were (mean ± SD) 5.82 ± 1.05 mm (right eye) during the PSF fixation task. To facilitate a descriptive and comparative analysis, Figure 2 presents the 15 randomly selected PSF image pairs that were used as stimuli in Phase 2 of the study. Each cell contains two images: A black‐on‐white image plane representation of the computed geometrical PSF on the left and its corresponding hand‐drawn PSF on the right. The geometric PSF represents the calculated light intensity pattern derived from wavefront aberration measurements with the contact lenses on the eye,^20^ whereas the participants' hand‐drawn PSF reflects their subjective perception of this light distribution.

There are several notable results evident in this set of PSFs. First, the PSFs exhibit considerable variation *between* the 15 image pairs in their overall structural characteristics, ranging from the radially symmetric patterns of some image pairs to more complex asymmetric distributions of other image pairs. For example, image pairs 10 and 14 exhibit strong radial symmetry with concentric circular patterns. By contrast, image pair 12 exhibits an asymmetric distribution with multiple three strong rays emanating from the upper left quadrant of the core. Other exemplar images have star‐like patterns with radiating arms (e.g., 1, 3, 4), diffuse patterns with distinct central features (e.g., 9, 13, 15), circular and radial elements (e.g., 2, 8, 11) and complex combinations of features (e.g., 5, 6, 7). Second, hand‐drawn versions *within* each PSF pair consistently capture the salient, characteristics of the corresponding geometric PSF, despite simplifying finer details of light intensity gradients. This finding demonstrates that participants can represent their perception of light sources with hand‐drawn illustrations that reproduce key structural features predicted in the geometric optics computed PSFs.

### Phase 2

#### Feature assessment and agreement

**FIGURE 3:**
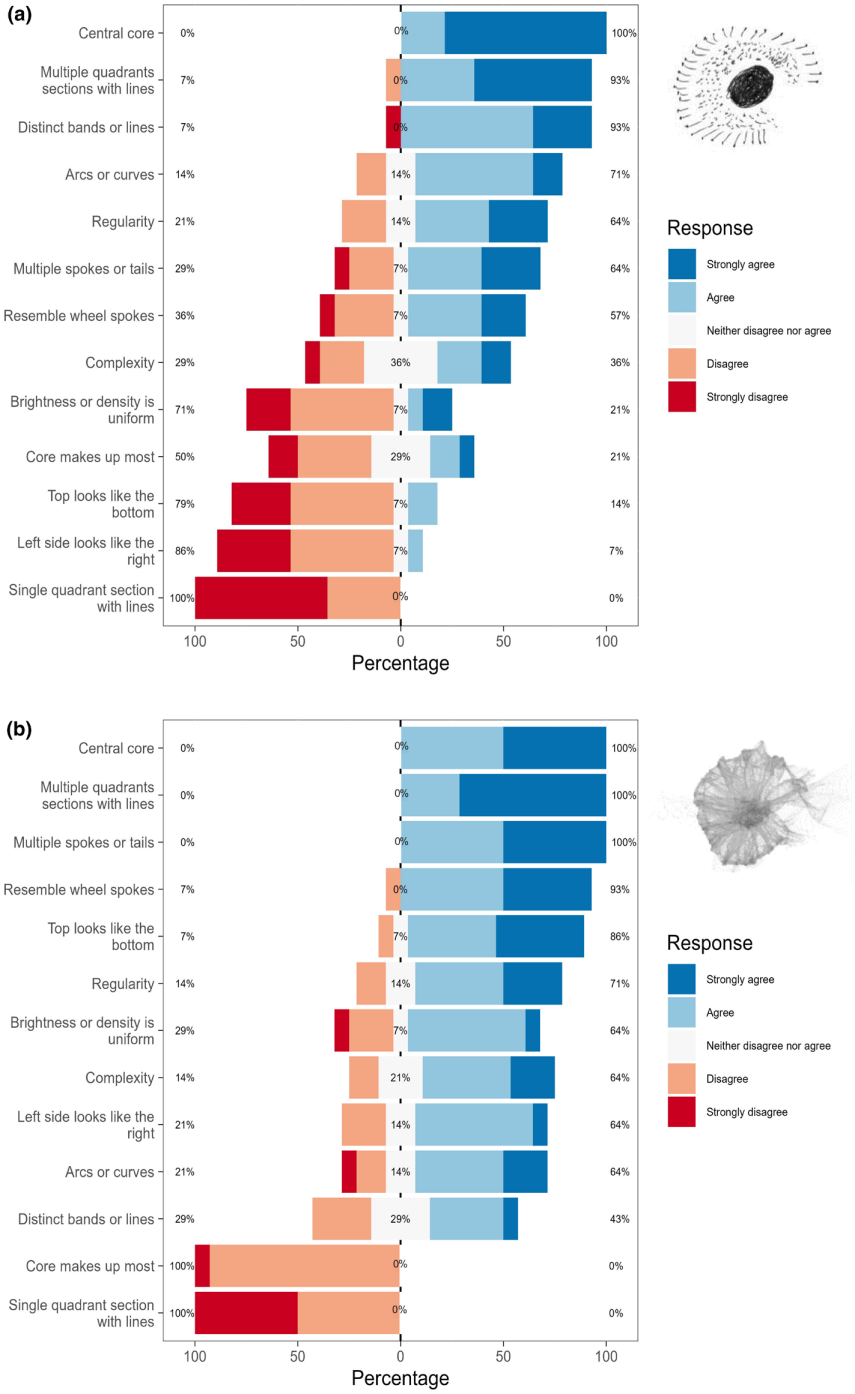
Comparison of Likert response distributions for an exemplar PSF image pair. Data for the participant drawn point spread function (PSF) are shown in the upper panel while data for the corresponding geometric version are shown in the bottom panel. Results are displayed separately for each feature with the percentages for the two categories of agreement and the two categories of disagreement combined. The percentage of responses in the neutral category are also shown in each row of the graph. In each panel, features are ordered such that the feature with the highest combined percentage agreement (light blue and dark blue bars) in the top row and the feature with the highest combined percentage agreement (light red and dark red bars) in the bottom row.

Figure 3 presents the feature agreement distributions of Likert responses for an exemplar PSF image pair. Results for the hand‐drawn PSF image are shown in the top panel while those for the corresponding geometric version are shown in the bottom panel. For each feature descriptor, the figure summarises the percentage of participant responses on a colour gradient that varies from dark red (Strongly Disagree) through dark blue (Strongly Agree). To facilitate interpretation, the numeric values listed to the left and right side of each bar represent the combined percentages of Strongly Disagree + Disagree responses and of Agree + Strongly Agree, respectively. Note that the feature descriptors in each panel are arranged vertically based on the strength of participant consensus. Those with the highest agreement percentages represent features that were most consistently identified in the PSF image and are positioned at the top of the panel. By contrast, descriptors with the highest percentages of consensus about the absence of the specified feature are positioned at the bottom.

As shown in Figure 3, the same two descriptors (i.e., ‘central core’, ‘multiple quadrants sections with lines’) had the highest combined *agreement* percentages for both the hand‐drawn (100% and 93%, respectively) and geometric (100% agreement for both features) versions of the PSF. All participants (100%) agreed that neither PSF had a ‘single quadrant/section with lines’. Importantly, the pattern of results seen in Figure 3 for this exemplar PSF pair is representative of the other image pairs evaluated in this task. Specifically, there was generally excellent agreement among participants about the features that were present in the PSF image and, as expected, less agreement about features that were not present in the image. Results for the group‐level consensus analysis using feature‐vector representations of the agreement percentages supported this finding. For example, the observed estimate of Yule's *Q* was 0.75 or greater for 11 of the 15 images. Further, strong positive correlations were observed between Yule's *Q* and four alternative association metrics evaluated in a sensitivity analysis. Specifically, Pearson product–moment correlations ranged from 0.40 between mutual information and Yule's *Q* to 0.99 between Phi and Kappa with an average of 0.76. Except for the correlation between mutual information and Yule's *Q*, all other correlations were statistically significant at *p* < 0.05.

#### Similarity judgements

**FIGURE 4:**
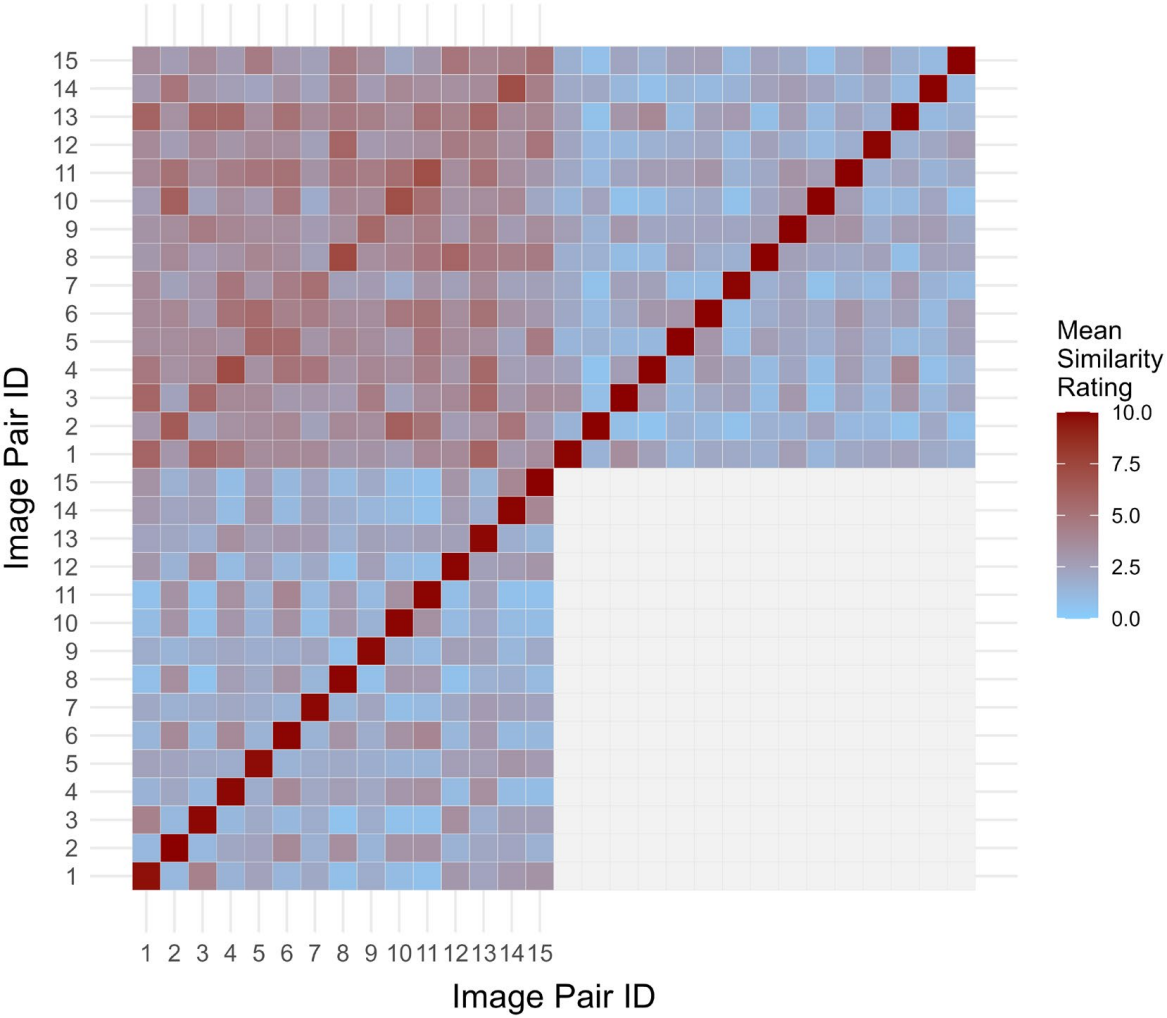
Heatmap representation of the group‐averaged pairwise similarity ratings. The results for each similarity rating task are displayed in a separate sub‐matrix of the map. Data for comparisons involving only geometric point spread functions (PSFs) (G2G) or only participant‐illustrated PSFs (D2D) are shown in the lower left and upper right matrices, respectively, while data for the drawn to geometric (D2G) comparisons are in the top left matrix. D2D, Drawn‐to‐Drawn; D2G, Drawn‐to‐Geometric; G2G, Geometric‐to‐Geometric.

In order to analyse quantitatively the qualitatively perceived similarity between computed geometric and hand‐drawn PSFs from Phase 1, a descriptive analysis was performed comparing the group‐averaged similarity ratings obtained in the three different stimulus conditions: drawn‐to‐drawn (D2D), geometric‐to‐geometric (G2G) and drawn‐to‐geometric (D2G). Figure 4 presents the mean similarity ratings as a heatmap, with each cell representing the average rating (*N* = 13) for specified image pairs. Results for G2G, D2D and D2G ratings are in the lower left, upper right and upper left 15 × 15 submatrix, respectively. The colour map legend provides the gradient of reference values for the mean similarity rating, with the lighter blue and darker red cells representing pairs that are rated the least and most similar, respectively. Diagonal elements in each submatrix serve as internal validation points, establishing the upper bound of the similarity scale. For the G2G and D2D tasks, data along the positive diagonals are based on the self‐comparison of a given PSF with itself and were therefore expected to yield the highest similarity ratings. For the D2G task, although the computed geometric and participant‐illustrated PSFs are naturally ‘paired’, there is not a direct pixel‐by‐pixel correspondence between the two images. Thus, in theory, the participant may never respond using ‘10 = identical’ on the rating scale.

The sample average statistics for the 15 self‐similarity comparisons are consistent with these expectations. Especially, the mean (SD; 95% CI) diagonal element rating is *M* = 9.88 (SD = 0.08; 95% CI: [9.84, 9.93]) for G2G, *M* = 9.98 (SD = 0.03; 95% CI: [9.97, 10.0]) for D2D and lower *M* = 5.99 (SD = 0.96; 95% CI: [5.46, 6.53]) for D2G. However, in contrast to the self‐similarity ratings, off‐diagonal ratings for the D2G task are, on average, greater than those observed in the G2G or D2D task. Especially, the mean (SD; 95% CI) off‐diagonal element rating is *M* = 2.03 (SD = 0.91; 95% CI: [1.85, 2.20]) for G2G, *M* = 1.88 (SD = 0.74; 95% CI: [1.74, 2.02]) for D2D and higher *M* = 3.78 (SD = 0.93; 95% CI: [3.60, 3.96]) for D2G. Our interpretation of this finding is that comparisons in the D2G task are more difficult (i.e., inherently noisier) than those in either the G2G or D2D tasks. Consequently, there is greater ambiguity when judging the similarity between a geometrically computed and drawn PSF resulting in greater use of the middle range of the 1–10 response scale.

**FIGURE 5:**
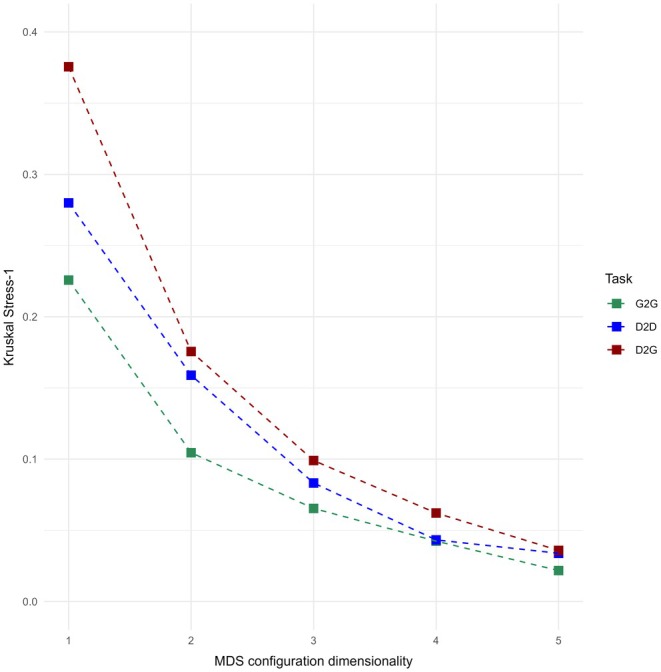
Kruskal Stress‐1 as a function of non‐metric multidimensional scaling (MDS) configuration dimensionality. A separate curve is shown for each PSF similarity rating task: Geometric‐to‐geometric (G2G, green), drawn‐to‐drawn (D2D, blue) and drawn‐to‐geometric (D2G, red) pairings. Based on goodness‐of‐fit guidelines, an MDS configuration in three dimensions provided an excellent fit (i.e., Stress‐1 < 0.1) to the observed data in each task.

Figure 5 compares values of the objective goodness‐of‐fit measure (Kruskal ‘Stress‐1’) as a function of MDS dimensionality, R, separately for each of the three paired comparison tasks, that is, G2G (green), D2D (blue) and D2G (red). Because this MDS stress measure is defined as the square root of the ‘residual sum of squares’, lower values indicate a better fit between the observed pairwise (dis)similarities and the predicted distance in the MDS configuration. There are several important aspects to the results. First, none of the stress values for *R* = 1 is less than 0.15. By convention, this implies that the true dimensionality, *R*_t_, of the data are greater than 1. Second, as expected, stress decreases monotonically as dimensionality increases. Following Kruskal's guidelines for stress interpretation, values above 0.20 indicate poor fit, 0.10–0.20 suggest fair fit and values 0.10 or lower represent excellent fit. From Figure 5, it can be seen that the value of Kruskal stress‐1 for D2G equals 0.38 for one dimension, 0.18 for two dimensions and 0.09 for three dimensions. Both the initial value at *R* = 1 and the substantial reduction in stress from one to two dimensions suggest that the one‐ or two‐dimensional solutions are insufficient to represent the data's underlying structure. The further decrease to 0.10 for three dimensions achieves what is considered an excellent fit. Although stress continues to slightly decrease with further dimensionality (from 0.06 to 0.04 for *R* = 4 and 5, respectively), the way interpretability changes from one dimensionality to the next becomes more complicated with minimal gain.^30^ Thus, based on both the minimal stress improvements at higher dimensions and visual analysis and interpretability of the configurations, a three‐dimensional representation of the data structure was selected for each task.

**FIGURE 6:**
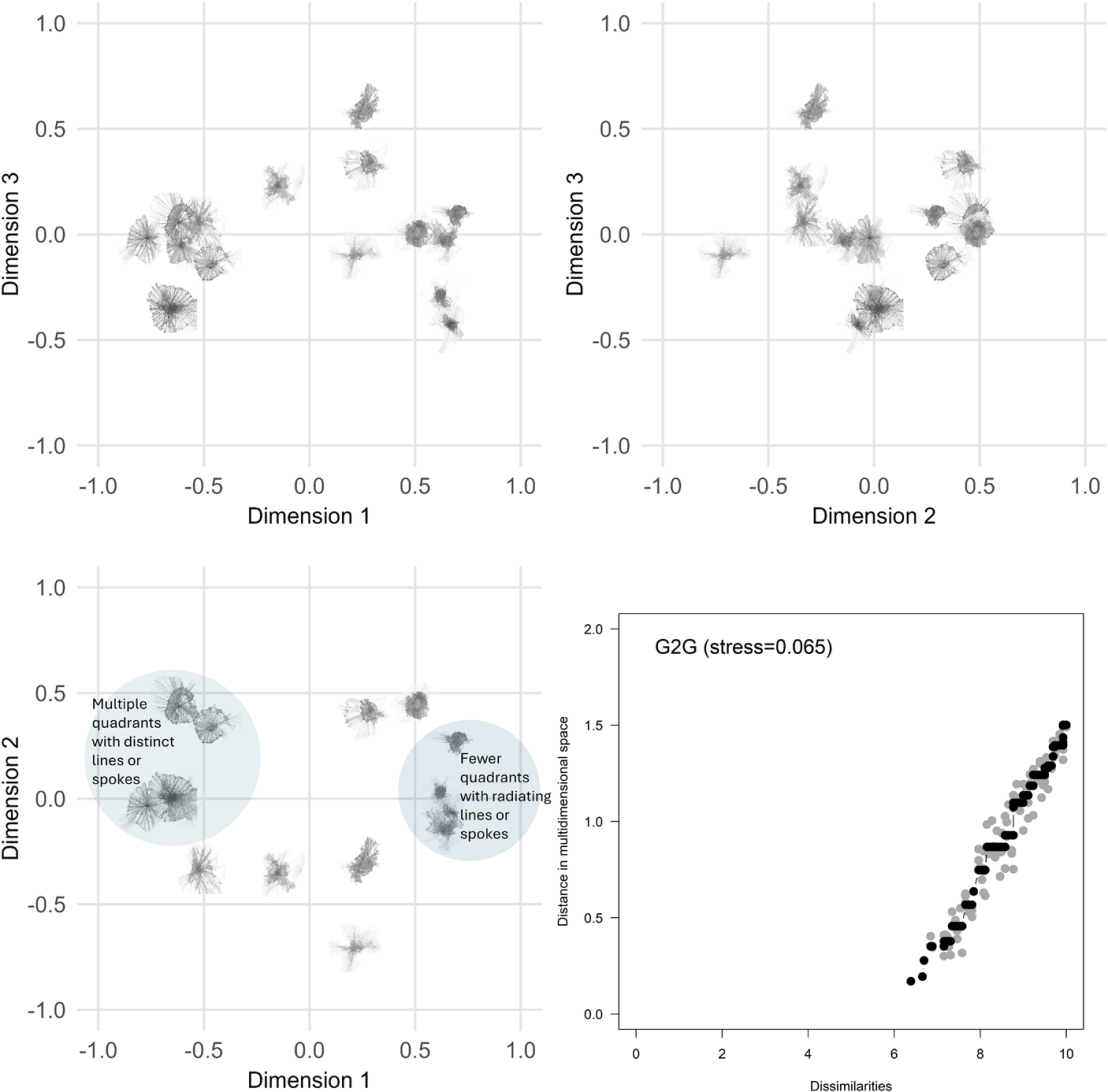
Three‐dimensional non‐multidimensional scaling (MDS) configuration for similarity ratings comparing geometric to geometric point spread functions (PSFs). Each scatterplot shows a different pairing of the dimensions. The lower left panel shows Dimension 2 (vertical axis) against Dimension 1 (horizontal axis), the upper left panel shows Dimension 3 (vertical axis) against Dimension 1 (horizontal axis) and the upper right panel shows Dimension 3 (vertical axis) against Dimension 2 (horizontal axis). A Shepard diagram with the fitted distances from the three‐dimensional MDS configuration plotted against the observed dissimilarities is shown in the lower right panel. This diagram includes an isotonic (i.e., least‐squares monotonic) regression line which is piecewise constant. Black data points represent predicted values and fall along the fitted regression line; light grey data points represent observed dissimilarities with their corresponding configuration distance. The stress value for the MDS configuration is proportional to the square root of the sum of the squared vertical distances between the observed distance and fitted regression line over all points. G2G, geometric to geometric.

**FIGURE 7:**
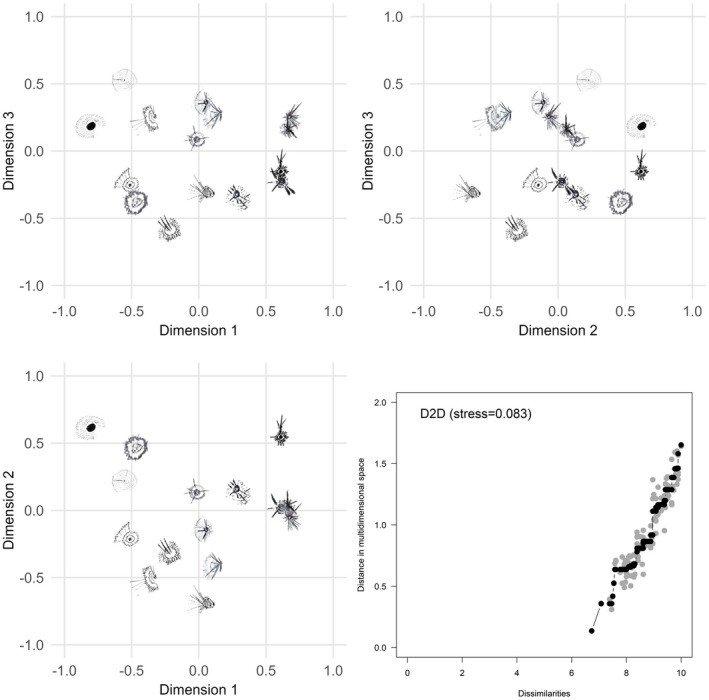
Three‐dimensional non‐multidimensional scaling (MDS) configuration for similarity ratings comparing participant drawn to drawn point spread function (PSF). Each scatterplot shows a different pairing of the dimensions. The lower left panel shows Dimension 2 (vertical axis) against Dimension 1 (horizontal axis), the upper left panel shows Dimension 3 (vertical axis) against Dimension 1 (horizontal axis) and the upper right panel shows Dimension 3 (vertical axis) against Dimension 2 (horizontal axis). A Shepard diagram with the fitted distances from the three‐dimensional MDS configuration plotted against the observed dissimilarities is shown in the lower right panel. This diagram includes an isotonic (i.e., least‐squares monotonic) regression line which is piecewise constant. Black data points represent predicted values and fall along the fitted regression line; light grey data points represent observed dissimilarities with their corresponding configuration distance. The stress value for the MDS configuration is proportional to the square root of the sum of the squared vertical distances between the observed distance and fitted regression line over all points.

**FIGURE 8:**
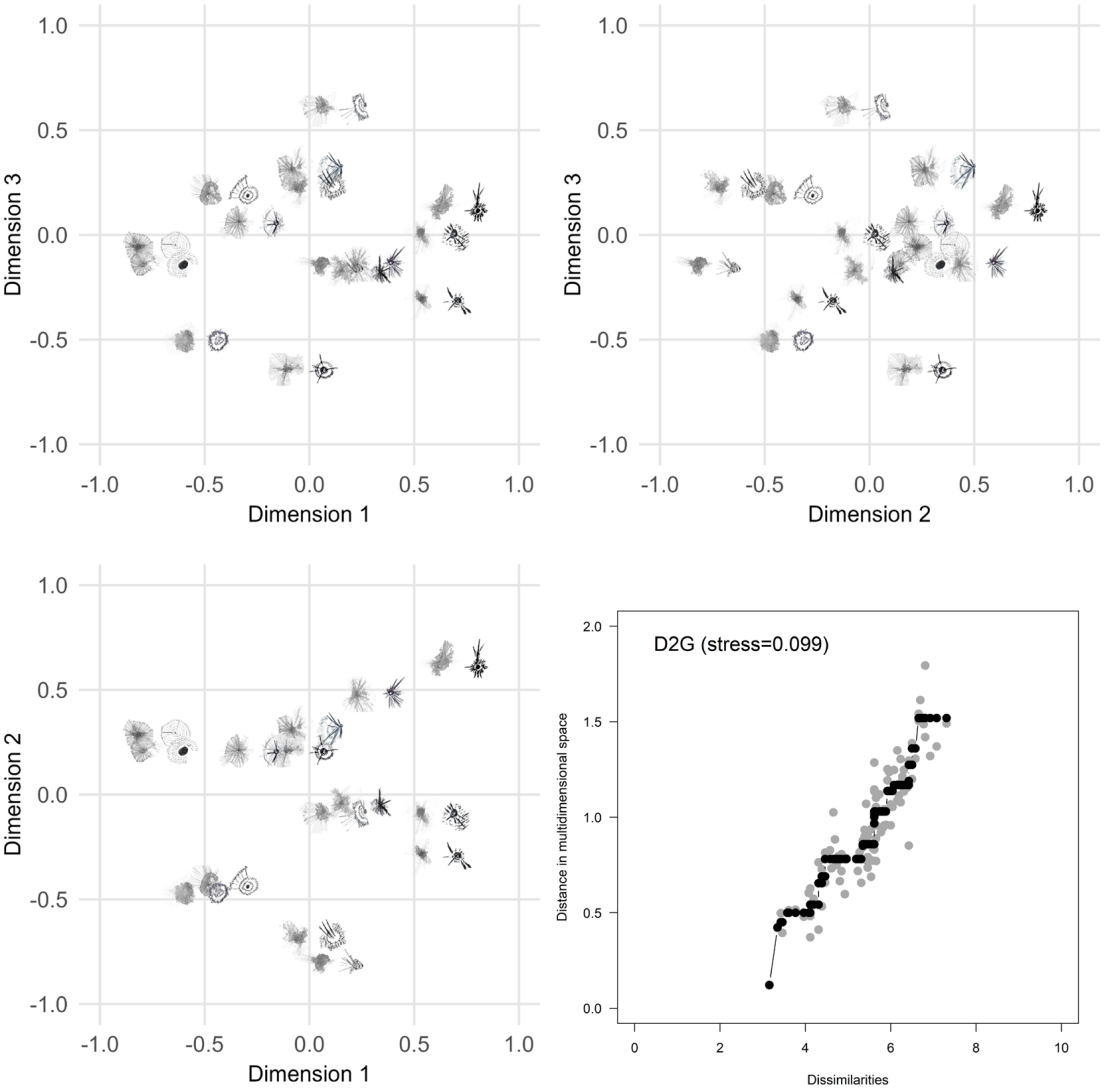
Three‐dimensional non‐multidimensional scaling (MDS) configuration for similarity ratings comparing participant‐drawn to geometric point spread functions (PSFs). Each scatterplot shows a different pairing of the dimensions. The lower left panel shows Dimension 2 (vertical axis) against Dimension 1 (horizontal axis), the upper left panel shows Dimension 3 (vertical axis) against Dimension 1 (horizontal axis), and the upper right panel shows Dimension 3 (vertical axis) against Dimension 2 (horizontal axis). A Shepard diagram with the fitted distances from the three‐dimensional MDS configuration plotted against the observed dissimilarities is shown in the lower right panel. This diagram includes an isotonic (i.e., least‐squares monotonic) regression line which is piecewise constant. Black data points represent predicted values and fall along the fitted regression line; light grey data points represent observed dissimilarities with their corresponding configuration distance. The stress value for the MDS configuration is proportional to the square root of the sum of the squared vertical distances between the observed distance and fitted regression line over all points.

Three‐dimensional MDS configurations for G2G, D2D and D2G are shown in Figures 6, 7, 8, respectively. Each figure provides a geometric representation of the spatial relationships between drawn or geometric PSF images alone (Figures 6 and 7, respectively) or for PSF pairs (Figure 8). To facilitate interpretation of the three‐dimensional MDS solutions, results are displayed as three two‐dimensional projections: x‐y, x‐z and y‐z planes, where Dimensions 1, 2 and 3 correspond to the x, y and z axes, respectively. In addition, each figure displays a Shepard diagram in the lower right panel that is based on the three‐dimensional MDS configuration.^31^, ^32^ This diagram is a scatterplot comparing two distance measures: The fitted distances from the multidimensional configuration (vertical axis) and the rank‐ordered observed dissimilarity ratings (horizontal axis), ranging from smallest to largest. The solid black line with black data points represents an isotonic regression line which, as expected for a non‐metric ordinal MDS solution, is a monotone, non‐decreasing function of the input dissimilarities. The observed stress value shown in the upper left corner of the panel is proportional to the root mean square vertical discrepancy between the grey data points and the regression line.

An attempt was made to interpret the dimensions using two visual inspection approaches, supplemented by the results from the feature assessment and agreement task. The first approach compared PSFs separated by large distances along each dimension, while the second examined proximal PSFs in different regions of the multidimensional space to identify common features. For example, for the G2G configuration shown in the lower left panel of Figure 6, PSFs positioned at large negative coordinate values on Dimension 1 had high participant agreement (>70%) on features including ‘central core’, ‘multiple quadrants with lines’, ‘multiple spokes or lines’ and ‘top/bottom symmetry’. Although there was also consensus about the presence of a central core, PSFs positioned at large positive coordinate values on Dimension 1 were generally considered as having a lower degree of symmetry and fewer quadrants with lines. Thus, from a contrast perspective, PSFs on the negative end (left) tend to be more compact and circular/radially symmetric, while those on the positive end (right) have more extended, branching structures with linearly radiating elements. This dimension could be labelled as ‘structural complexity’ or ‘branching versus compactness’ Note that the projections onto Dimension 1 remain relatively consistent with those in the upper left panel which plots Dimension 3 against Dimension 1. Next, looking at the contrast between positive and negative values on Dimension 2 of the G2G configuration, PSFs at the positive end tend to have more distinct, separated lines, bands or spokes (highlighted by light blue‐filled circles), while those at the negative end appear more unified in their structure. Thus, this dimension could represent ‘numerosity’ or ‘element separation’. For Dimension 3 (shown on the vertical axis in the upper left and upper right panels), the contrast appears to relate to density or complexity of internal details within the PSFs. Ones at the positive end tend to have more intricate internal patterns or details, while those at the negative end are simpler or sparser in their internal structure. This dimension might represent ‘internal complexity’ or ‘detail density’. Using the clustering‐based approach to interpret the projections shown in the lower left panel, there is a grouping of PSFs located in the lower left quadrant that share characteristics of having a circular pattern with a central core and relatively simple internal structure consisting of multiple radial lines of similar extent. This supports the dimensional interpretation for Dimensions 1 and 3.

Similar patterns in the MDS configuration and 2D projections were observed for D2D comparisons (Figure 7), albeit with slightly increased overall stress (0.083 vs. 0.065 for G2G). Using a dimensional contrast approach, there was consensus that PSFs at more negative coordinates of Dimension 1 have a salient central core, multiple quadrants with lines or spokes and exhibit regularity and/or symmetry. PSFs at positive coordinate values on Dimension 1 have distinct lines or bands and vary with respect to the degree of symmetry. Lastly, although the MDS configuration for D2G (Figure 8) resulted in the highest stress (0.099), the spatial relationship among the PSF pairs is generally consistent with both G2G and D2D configurations. Specifically, in addition to dimensional contrast in terms of multiple quadrants, lines or spokes, and symmetry/regularity, clusters of PSFs were seen with similar features separated by small distances in different regions of the space.

**FIGURE 9:**
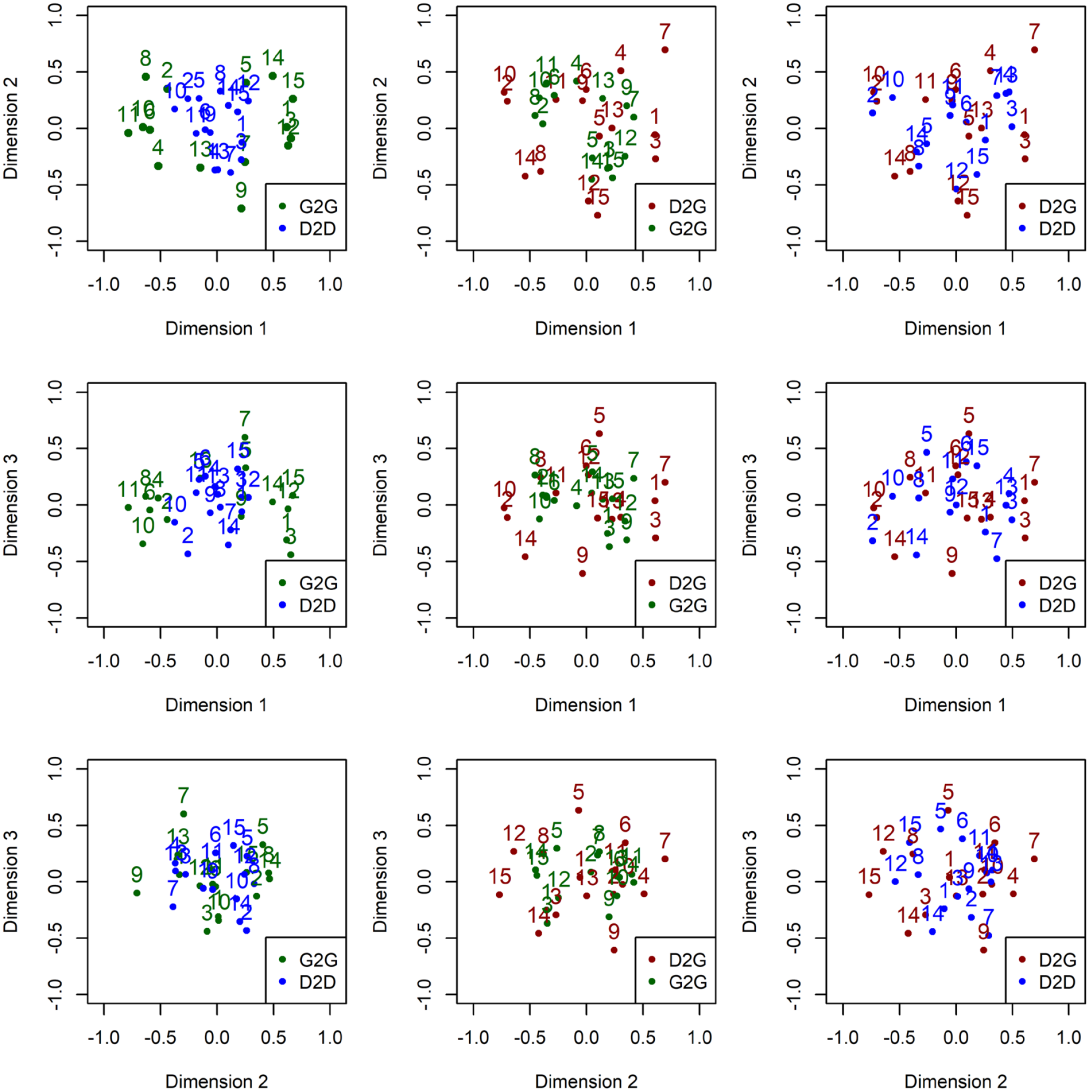
Procrustes transformation analysis comparing non‐multidimensional scaling (MDS) configurations for G2G with D2D (left panel), D2G with G2G (middle panel), and D2G with D2D (right panel). The analysis removes differences such as rotation, translation and dilation without changing the fit (stress) of the MDS solution. D2D, Drawn‐to‐Drawn; D2G, Drawn‐to‐Geometric; G2G, Geometric‐to‐Geometric.

A Procrustes transformation analysis provided evidence for the similarity across the 3D MDS configurations. Congruence coefficients were 0.90 (G2G vs. D2D), 0.94 (D2G vs. G2G) and 0.96 (D2G vs. D2D).^33^, ^34^ In addition, strong positive correlations were observed between D2G and G2G (*r* = 0.69) and between D2G and D2D (*r* = 0.79), while a moderate correlation (*r* = 0.55) was found between G2G and D2D. Figure 9 plots the Procrustes‐transformed solutions for Dimension 1 and Dimension 2 (top), Dimension 1 and Dimension 3 (middle) and Dimension 2 and Dimension 3 (bottom). Separate plots are shown for G2G with D2D (left column), D2G with G2G (middle column) and D2G with D2D (right column). Taken together, the underlying spatial relationships between PSF images are well preserved across the different MDS configurations. Thus, the congruence between MDS configurations indicates the solutions were robust with respect to task differences and/or potential difficulties comparing similarity between hand‐drawn and geometrically computed PSFs.

#### Image‐matching

The image matching results for two participants provided additional validation of the perceptual similarity between the computed geometric PSFs and their corresponding participant‐drawn PSFs. Although the number of correct matches differed between these participants, the likelihood that the observed performance was due to random guessing was extremely low. Specifically, the number of correct matches (*n*_*C*_), the percentage of correct matches out of 15 (%) and the probability of observing *n*_*C*_ correct matches by guessing (pr(*n*_*C*_|guess)) were *n*_*C*_ = 10 (67%) with pr(*n*_*C*_|guess) = 1.04 × 10^−6^ for the first participant and *n*_*C*_ = 8 (53%) with pr(*n*_*C*_|guess) = 7.30 × 10^−5^ for the second participant. Thus, the matching performance for both participants provides further direct evidence for strong alignment between the computed geometric PSFs and their hand‐drawn counterparts.

## DISCUSSION

This study was designed primarily to compare two distinct approaches for representing retinal image quality: computational geometric point‐spread functions (PSFs) and participant‐drawn subjective PSFs. In the first phase of the analysis, geometric PSFs calculated from wavefront vergence maps provided an ‘objective’ measure of how the eye fitted with a contact lens responds to a point source of light. Participant drawings of their perceived visual response to that point source acquired concurrently provided a ‘subjective’ measure. Consistent with previous descriptive/qualitative reports,^10^, ^35^ excellent correspondence in shape was found between the geometric and hand‐drawn PSFs, both in terms of overall shape (e.g., circularity, orientation) and specific details (e.g., ‘starbursts’, asymmetry in location of radial lines). In the second phase of the analysis, data from three different psychometric tasks were used to evaluate quantitatively the relationship between the geometric and hand‐drawn PSFs. For example, Likert rating scale data were used to assess the inter‐participant agreement about the feature descriptors characterising the two PSF representations. Results revealed a high degree of consensus in feature identification across individuals. Importantly, this finding provides strong justification for the use of participant‐drawn images as an easy, reliable method for capturing and quantifying retinal image quality. To gain further insight regarding the underlying dimensions of the PSF feature space, pairwise similarity ratings data were analysed using non‐metric multidimensional analysis. Although the ‘true’ underlying configurations in some dimensionalities are unknown, excellent model fits were achieved in three dimensions for all comparison types, that is, pairings of geometric PSFs only, pairings of hand‐drawn PSFs only and pairings of geometric PSFs with hand‐drawn PSFs.

Practitioners face several challenges when optimising corrective/treatment lenses, particularly when fitting multifocal or dual focal contact lenses (MFCL) which is further complicated by the disparity between objective measures and subjective patient‐reported visual symptoms. Objective aberrometry with computational optical techniques, however, can successfully predict what wearers of multifocal SCL subjectively appreciate and describe. Specifically, shape characteristics of computationally modelled PSFs closely matched those in subjectively hand‐drawn PSFs, indicating that the optical modelling captured accurately the visual blur experienced by MFCL wearers.

Of course, the current results only assessed monocular image quality at distance, so further work is needed to explore the impact of viewing distance (e.g., during near viewing), specific zone geometries and how binocular perception can be predicted by the combination of the optical signatures of the right and left eyes. Additionally, further work is necessary to compare quantitatively the agreement in computationally predicted PSF size to that perceived by the participant. Nonetheless, the current findings demonstrate that computational optics can predict on‐eye lens performance reliability, opening opportunities for streamlined and improved lens design (e.g., efficiently test and iterate) and patient fitting/prescribing (e.g., optimise lens optics for an eye).

## AUTHOR CONTRIBUTIONS

**Pete Kollbaum:** Conceptualization (equal); formal analysis (equal); investigation (equal); methodology (equal); validation (equal); visualization (equal); writing – original draft (equal); writing – review and editing (equal). **Dawn Meyer:** Conceptualization (equal); formal analysis (equal); investigation (equal); methodology (equal); validation (equal); visualization (equal); writing – original draft (equal); writing – review and editing (equal). **Nitya Murthy:** Conceptualization (equal); formal analysis (equal); investigation (equal); methodology (equal); validation (equal); visualization (equal); writing – original draft (equal); writing – review and editing (equal). **Javier Gantes‐Nunez:** Conceptualization (equal); formal analysis (equal); investigation (equal); methodology (equal); validation (equal); visualization (equal); writing – original draft (equal); writing – review and editing (equal). **Matt Jaskulski:** Conceptualization (equal); formal analysis (equal); investigation (equal); methodology (equal); validation (equal); visualization (equal); writing – original draft (equal); writing – review and editing (equal). **Martin Rickert:** Conceptualization (equal); formal analysis (equal); investigation (equal); methodology (equal); validation (equal); visualization (equal); writing – original draft (equal); writing – review and editing (equal).

## CONFLICT OF INTEREST STATEMENT

PK: research: Alcon, CooperVision, Inc., EssilorLuxottica, Hoya, Johnson and Johnson Vision, SightGlass Vision; PK: consultant EssilorLuxottica, SightGlass Vision; DM: none; NM: none; JG: none; MJ: employee/owner VisionApp; MR: consultant: CooperVision, Inc. SightGlass Vision.

## CLINICAL TRIALS REGISTRATION

Because this study was intended to test the early feasibility of the device and/or basic research concept(s) being studied, it was not considered an applicable clinical trial for clinical trials registry (ClinicalTrials.gov or equivalent). As such, this clinical trial was not registered.
